# Ignaz Philipp Semmelweis and His Times
*From an Address to the Bath Clinical Society, 13th February, 1959.


**Published:** 1960-03

**Authors:** Frederick Kohn

**Affiliations:** Late Medical Superintendent of St. Martin's Hospital, Bath


### IGNAZ PHILIPP SEMMELWEIS AND HIS TIMES*

BY

FREDERICK KOHN, M.D., PRAGUE

*Late Medical Superintendent of St. Martin's Hospital, Bath*

I have chosen Ignaz Philipp Semmelweiss as the subject tonight for two reasons. First, I have found that the name of Semmelweis is not very well known and I hope to show that it is a name worthy to be remembered, and secondly he belonged to the so-called Second Viennese School which once was famous all over Europe, and I thought you might be interested to glimpse at those times.

Let us go back to the end of the eighteenth century when considerable changes were made in the life of the Austrian people, especially under the Emperor Josef II (1780–90). He introduced compulsory education from six to thirteen, abolished serfdom (1789) and restricted the power of the Church, abrogating many clerical privileges and closing certain monasteries particularly those of the Jesuits. Serfdom was restored by his brother Leopold who reigned only two years and was succeeded by his son Franz II, the last Holy Roman Emperor and the First Emperor of Austria. All the internal problems were, however, overshadowed by the wars with France, which began in 1792 and lasted with interruptions until 1814. These brought to Austria a series of disasters: Austria lost her possessions in Italy and the Rhineland, Vienna was twice occupied by French armies and in 1806 Napoleon himself became universal Emperor. The prevailing instability made Franz II extremely conservative; he resisted change, popular initiative or indeed any movement in political affairs; his resistance to change was such that he would not even alter the reforms made by Josef II, although he wholeheartedly disagreed with all reforms introduced by his predecessor. When Franz died in 1835 he was succeeded by his son Ferdinand almost an imbecile.

In this unstable and rather confused atmosphere a very powerful Civil Service and Administration developed. Its head was the very able statesman Prince Clemens Wenzel Metternich, who had the complete confidence of the Emperor and the court in a wider sense. The many rules and regulations were exactly recorded and codified and the saying was:

"Quod non est in actis, non est in mundo".

Even the University came under control of the Ministry and this affected the academic Senate and the professors directly.

A professor regius ordinarius once confirmed by the Emperor was all-powerful in his own department and could not be removed unless he was offered a chair at another University.

Yet with this rigid imperial system the social life of Vienna offered a very different picture. Here were living or had recently lived Mozart, Haydn, Schubert and Beethoven and a circle of artists that included the painter Moritz Schwind, the poet Bauernfeld and Grillparzer, the singer Vogl and many outstanding musicians, among them the violinist Kreutzer to whom Beethoven dedicated the Kreutzer Sonata. If the regime was dour and gloomy, the population was gay and cheerful. Indeed, one can say that this ironic mixture persisted in Vienna until the end of the Empire. It was in contrast to that in Germany. The contrast was summarized at the end of the First World War when the people in Berlin said: "the situation is serious but not hopeless" and those in Vienna said: "the situation is quite hopeless but not serious".

*From an Address to the Bath Clinical Society, 13th February, 1959.

The hospital which was the scene of the work and suffering of Semmelweiss was the Lying-in Division of the General Hospital of Vienna. It had been founded by the Empress Maria Theresia in gratitude to Van Swieten, her Dutch doctor and founder of the greatness of the Viennese Medical School. The lying-in Division was found necessary because of the immense number of illegitimate children. This in turn was caused by a ridiculous law that forbad marriage to illiterates. Nor did this affect only the poorer classes for even among the well to do, the number of illiterates was considerable. The arrangements for admission to the Lying-in Division shed a sombre light on the state of society

The early figures of the Viennese School are an impressive line. After Van Swieten and Auenbrugger, who in the eighteenth century introduced percussion as a means of diagnosis and prognosis came the pathologist Johannes Wagner, a Bohemian by birth; he was one of the first who drew his conclusions from the changes in the affected organs and not from the symptoms alone. His personality drew men like Rokitansky, Kolletschka, Schuh ahd Skoda to Vienna. He died at 33 years of T.B. He was soon succeeded by Rokitansky of Koeniggraetz in Bohemia.

Rokitansky started medical study in Prague in 1822, where the young Purkinje was one of his teachers, but he went to Vienna in 1824 and graduated there in 1829. He became an unpaid assistant to Wagner whom he succeeded, first as professor extra-ordinarius, and in 1844 as ordinarius. Rokitansky is well remembered as the most widely experienced of all morbid anatomists; his series of post-mortems in 1829 begins with the number, 4,781 when he retired in 1875 (31st August) the serial number is 64,571. In addition to these he made 25,000 forensic medical post-mortems. It needed a great working capacity to sort out and adequately to present this enormous material.

Rokitansky's teaching then was that the great variety of diseases could be explained by a great variety of the mixture of the blood (Humoral pathology). These views were quickly set aside when Virchow's Cellular-pathology (*Omnis cellula, e cellula*) became known. Rokitansky had to work in two small rooms which could neither be heated nor ventilated. There wasn't even enough light and to see properly it was necessary to climb on to a chair placed on a table near the window. Not until 1862 that a new building was completed and over its door was fixed a quotation from Morgagni: *Indagandis sedibus et causis morborum*" ("For investigating of the seats and causes of diseases").

Rokitansky never practised medicine himself, but he made himself available when in 1831 and again in 1832 a severe epidemic of cholera of which more than 5,000 people died in Vienna. In politics he was a liberal, in those days considered outrageous, but nevertheless he was created a Counsellor (Hofrat,) a baron and a member of the *Herrenhaus* (House of Lords). He died three years after his retirement on the 23rd July, 1878.

The second Viennese School was completed by two great personalities, Skoda, and a little later, Hebra. Skoda was born in 1805 in Pilsen, again in Bohemia, the son of a locksmith. He began to study medicine at Vienna University in 1825, where, like his friend Rokitansky, he had to support himself by coaching younger students. He took his degree in 1831 and became an unpaid assistant at the General Hospital in 1832. In 1833 he became a paid assistant and so remained until 1839. In 1840 he became Head of the Department for Chest Diseases.

Medicine in general was then much under the influence of the philosophy of the time and might have so remained but for Skoda and those around him. We may recall Hahnemann's homoeopathic doctrine: (1755–1842) *Similia similibus curantur*, a dictum very fashionable in those days. Skoda's new findings developing the work of Auenbragger and were publicized by Franz Schuh. They were treated by his contemporaries with derision. It was a gibe of a professor von Hildebrand "I am fond of music, but have never heard a pneumonia play the fiddle". When Skoda retired in 1871 he said:

"now it is up to the microscope and chemistry". Skoda's aim was to concentrate on the physical methods of examination in diagnosis, a prognosis.

Ferdinand Hebra was an assistant of Skoda. He showed great interest in the diseases of the skin. At that time all sub-departments or, as we now call them, specialities, came under the supervision of the professor of general medicine. Skoda offered Hebra the opportunity to take over the care and supervision of the skin case of his department and thus Hebra became one of the founders of Dermatology. Prominent among Hebra's pupils were Kaposi and Philip Josef Pick. Hebra linked dermatology to Pathology, influenced by Rokitansky and his first paper was on scabies. Among other personalities of the Second Vienna School we may recall the anatomist Hyrtl, the physiologist Bruegge, the physician Oppolzer, the surgeons Schuh and Dummreicher, followed later by the eye specialist Arlt and the greatest of all, Billroth.

Contrasted with these pioneers was Johann Klein, professor of obstetrics, he was a narrow minded official, obedient to his superiors but impatient with his subordinates, unintelligent but very powerful, because he was not only regius professor and therefore irremovable, but also director of the General Hospital with its entire administration in his hands. No changes were possible without his consent, every detail had to come before him and was decided by him. Not only had he the final say in dealing with hospital problems as such, but he also could transfer, admit or dismiss both lay and medical staff. Thus, for example, he transferred Skoda before he became professor, from the General to a Mental Hospital in one of the suburbs because he disagreed with Skoda's use of the stethoscope. Only when Skoda's views were recognized not only in Vienna but also further afield was Klein forced to recall Skoda. We shall meet again the ill-famed Klein.

These were the men whom Semmelweis found when he came as a student to Vienna in 1837. He was born in Budapest on the 1st of July, 1818, the son of a shopkeeper. When he came to Vienna he was looked on by the citizens as an alien from an unfriendly land for the awakening of nationalism had begun to cause tension with Hungarians who sought independence. Semmelweis, a rather poor student, found himself lost and lonely in a strange and unfriendly world. For a few months he studied law; then changed to medicine. Rokitansky accidentally became aware of the poor and undernourished looking young man and invited him to stay at his house. After a break of several months in Budapest, owing to the death of his mother, Semmelweis returned to Vienna and, after qualifying, became assistant at the obstetrical clinic.

The obstetric clinic was divided into two parts, one was used for the training of medical students and was under the immediate direction of Klein, while the other was used for the instruction of midwives and was in charge of Bartsch. Semmelweis was at once shocked by the enormous proportion of deaths in Klein's clinic, amounting to 10 per cent. at least, sometimes rising to 20 per cent., while the number of deaths occurring on the second clinic was much smaller, fluctuated between 3 per cent and 4 per cent. The authorities, who considered puerperal fever as a peculiar form of epidemic disease, blamed all sorts of atmospheric, telluric, cosmic, and miasmatic influences for the mortality in Klein's clinic. Again they made overcrowding responsible and it was even said that the sense of modesty was too frequently violated because the male students came to examine the women. In the end foreign students were blamed and it was maintained that they were rough with the patients before and while they were in labour. This latter accusation went so far that the number of foreign students was restricted and when there ensued no reduction in the number of deaths, foreign students were excluded from Klein's clinic.

This state of affairs became of course known among the people and the women tried to avoid the first clinic. The two clinics admitted on alternate days and there was very often a fight not to be admitted to the "Death House" as it came to be named. Semmelweis writes: "One could see heart-rending scenes when patients on their knees asked weeping, to be discharged again after they found themselves by mistake in the first clinic while they had expected to be or had booked for the second. Women in childbed with an uncountable pulse rate, enormous meteorism, dry tongue, gravely ill with puerperal fever declared only a few hours before their death that they were quite all right, to avoid treatment, which they knew was but the fore-runner of death". Semmelweis was desperate and could find no explanation for the enormous proportion of deaths in the first clinic while that in the second clinic remained around 3 to 4 per cent. The findings of the Royal Commission, which had blamed the foreigners, bad ventilation, dirty linen etc., in 1846 were to him not a sufficient explanation. He noticed, however, that all women who had been in labour with a slow first stage were taken ill during the 24–36 hours after delivery and died very quickly and that the children also died soon afterwards. Yet a woman who was delivered in the street had a much lower morbidity—and mortality rate.

In complete helplessness Semmelweis abolished the posterior position in labour used in the first clinic and introduced the lateral position just because it was practised in the second. Still the mortality rates remained the same. Depressed and exhausted, he went in the early spring of 1847 to Venice to forget the horrors at the clinic. When he returned after three weeks to Vienna he heard to his dismay that his friend Professor Kolletschka had died. Kolletschka had been doing most of the post mortems in connection with forensic medicine and, while performing a post-mortem, cut his finger. An infection set in with severe lymphangitis and lymphadenitis and death occurred within a few days. The findings at the post-mortem were: bilateral pleurisy, pericarditis, peritonitis, meningitis, metastases in one eye—very much as he had seen in so many of his patients who had died of puerperal fever.

It now became clear to Semmelweis that there must be a direct connection between the death of Kolletschka and the deaths of hundreds of his patients, in fact, that he had died of puerperal fever. The medical students coming from doing post-mortems under supervision of the Professor or his assistants, and then coming directly to the Lying-in Division and examining the women in labour, *must be the carriers* of some sort of poison which was causing the puerperal fever. He came to the conclusion that particles from a corpse, were adherent to the fingers of the students or the doctors passing directly from post-mortem room to the women in labour and there examining patients without properly washing their hands. It was, he believed, their fingers that were responsible for the introduction of the material which carried the fever that killed his patients.

To prove the correctness of this view Semmelweis, in 1847, made it a rule that all students and doctors must wash their hands first with soap and water and then in a solution of Calcium Chlorate before examining patients. The immediate result was a startling change in the mortality rate of the first clinic. From more than 12 per cent. it sank, to the same level as that of the second clinic.

In October 1847, a patient in labour and suffering from a carcinoma of the uterus was admitted to bed number one where the round always started. After she had been examined, the students and doctors washed their hands but with soap and water only and of 12 women who were delivered at the same time, 11 died of puerperal fever. This was for Semmelweis proof enough, that not only disentegrating organic material from a corpse but putrefying organic material from the living is capable of producing puerperal fever. Soon after there was a like experience with a woman suffering from a septic knee, and his views were reinforced.

Just at this time Professor Klein, who seems to have disliked Semmelweis from the beginning, began to make difficulties, which in the end forced Semmelweis to leave Vienna. Without giving any reason he ridiculed Semmelweis before the medical students by pointing out that Semmelweis' views were not sufficiently tested; and he gave orders that no further Calcium Chlorate should be supplied to the wards, as the cost was too high. As a matter of fact it was very cheap, and, as Semmelweis was determined to use it, he provided the necessary money himself.

But Klein was not beaten; he now told the students that the ideas of Semmelweis were useless, and that he had no objection if the orders of Semmelweis on washing were not observed. Meantime, the views of Semmelweis had been favourably mentioned in two articles by Hebra in a Viennese journal (December 1847 and April 1848) and Haller, one of the physicians also spoke of Semmelweis's success in a lecture and expressed himself favourably concerning the practical consequences of Semmelweis's doctrine. The first full exposition was that of Skoda in a lecture delivered in the Viennese Royal Academy in 1849. In May 1850 Semmelweis himself developed his views on the cause of puerperal fever and rejected the idea of direct contagion. Not every patient suffering from puerperal fever will transmit the condition to a healthy person, and on the other hand, a healthy person can become infected with puerperal fever from a person who herself is not suffering from the disease. As a comparison he says: "every person suffering from small-pox is capable of transferring the illness to a healthy person, and a healthy person can only be affected with the same disease by a person suffering from smallpox. No person will become infected with smallpox from a person suffering from a carcinoma of the uterus. When puerperal fever takes its course under conditions where no organic material disintegrates or putrefies, then puerperal fever is not transferable to a healthy person. If on the other hand disintegrating organic material is produced in the course of puerperal fever, as for instance a septic endometritis, then puerperal fever is transferable to a healthy person. After death it is transferable from any corpse, but then it is the degree of decomposition which matters. Puerperal fever may originate from diseases which by themselves are not puerperal fever, for instance erysipelas, carcinoma of the uterus, etc. Therefore, he concludes, puerperal fever is not a contagious disease, but it is transferable to a healthy person by decomposing organic matter. Such a transfer can start anywhere outside or inside the human body where continuity of the normal integument, epidermis or epithelium is broken.

It should here be recalled that along with the high mortality rate of the mother at Klein's clinic was the perhaps still higher death rate of the new-born, who after the mothers' deaths were transferred to a foundling asylum. There too the post-mortem findings were identical with those of their mothers: Bednar, the physician to the asylum reported in 1850: "septicaemia in the newborn has now become very rare and for that we have to thank the discovery of Semmelweis who found the cause and means of prevention of this murderous disease". Most Continental obstetricians and pathologists still believed that a *genius epidemicus*, *i.e.* a cosmic or telluric miasma caused puerperal fever and the doctrine of Semmelweis was rejected by most. Their view was rigorously expressed in 1858 by the Academy of Medicine in Paris and in 1861 by the "Versammlung deutscher Naturforscher" (Congress of German Scientists) at Speyer. Dubois in Paris, one of the highest medical authorities in France said in 1858: "The views of Semmelweis generally accepted in Germany and England, that the transmission of puerperal fever may take place by the blood, by various discharges from the diseased or by decomposing organic material, has been proved wrong, and perhaps this doctrine is by now completely forgotten at the place where it originated. But of course that does not mean that one should not be careful and take all precautionary measures but I do not think that all the contagious qualities are either so constant or so persistent or vigorous as was alleged in the reports. If it were really so serious, the whole staff of the hospitals would have to be kept in strict quarantine, otherwise the public would constantly be in great peril. Therefore it is due to the public to reduce the exaggerated assumptions to their proper level. In many women before delivery these are conditions which favour the development of puerperal fever, as can often be seen in private practice and in hospitals. In the latter many women, pregnant or in labour, arrive with evident symptoms of puerperal fever which very often takes a rapid course." Semmelweis answered "My teaching is slandered in Vienna but not forgotten."

Another opponent was the pathologist Virchow in Berlin who called Semmelweis a speculator and in 1858 still made the weather responsible for puerperal fever.

Semmelweis called him a bad pathologist. In 1863 Virchow was still saying "The main factor in the origin and the spread of puerperal fever is a predisposition of the individuum for the development of diffuse and malignant types of inflammation and therefore the development of puerperal fever can happen even without a contagium". And again "For many years while I have daily handled corpses and pathological specimens I have been at the same time, in my wards at the Charité, and have treated women in child-bed with the very best results".

The most violent opponents of Semmelweis were, however, his fellow gynaecologists. It would take several articles to quote all the opponents who made life for Semmelweis hard indeed. One of the most emphatic was Scanzoni, Professor of Obstetrics in Wurzburg, and previously in Prague and there developed a quarrel between the two which went on for years; Semmelweis called him "the Nero of Medicine".

Semmelweis continued to try very hard to convince his chief Klein, that the measures taken by him were effective and that other obstetricians in Vienna and Prague as well as in Germany, France and England were sending him favourable reports. The relationship between the two grew worse and in 1849 Semmelweis decided to resign his post and to ask for right of a free teacher at the University, which he hoped would be granted him with the help of his friends Skoda, Rokitansky and Hebra. But he reckoned without Klein, whose influence with the authorities was so strong that the request of Semmelweis was bluntly rejected without reason given. A year later he made a second application and this time it was granted but with the restriction that he was allowed to discuss only the *theory* of obstetrics and that no practical demonstration was allowed.

Tired and disappointed he left Vienna for Budapest in 1850. Soon after he met his colleagues at a discussion of the Budapest Medical Society and his theory was of course discussed and straightaway attacked. He was told that there was a serious outbreak of puerperal fever at the St. Rochus Hospital where there are no students to be blamed. When he went there the next day he found that the surgeon in charge of the hospital was not only looking after his surgical and gynaecological patients, but he was at the same time also pathologist of the place and he and his assistants did all the post mortems! In 1851, Semmelweis was appointed unpaid Honorary Primarius and chief of the gynaecological and obstetrical unit at the St. Rochus Hospital, a post he held until 1857. In 1855, after the death of Professor Birly, he was appointed to the chair and became professor ordinarius at the University. The condition of the buildings of the St. Rochus—and the University Hospital in Budapest were if possible even worse than in Vienna.

The remaining years of his life were spent in Budapest, where he tried hard to convince his colleagues and pupils of his views. He proded their truth by reducing the death rate of puerperal fever to a hitherto unknown low figure; yet he still met fierce opposition and he became a tired, exhausted man, often suffering from insomnia and taking refuge in hard work. At the end of 1864 a condition developed which for a time kept him from work. He recovered but, in 1865 it was found necessary to admit him to a private asylum in Dobling near Vienna, where Hebra had found a room for him. He died a few days later of a septicaemia from a small wound on his finger, acquired at an operation which he performed in one of his last lucid moments. The post-mortem findings (recorded on the death certificate) were exactly the same as those of the victims of puerperal fever. He was buried in Vienna. Thirty years later his body was brought to Budapest and buried there.

As we have seen, the situation on the Continent was as black as it could be before Semmelweis took up his fight against the mortal danger of childbed fever. The situation in England and in Ireland was very different. The main reason for this was: firstly that puerperal fever was regarded there as a contagious disease and strict precautions were taken to prevent its spread; and secondly, that long before Semmelweis emitted his calls of warning the same ideas had been expressed there and led to the necessary precautions. These included the washing of the hands in antiseptic liquid. Charles White of Manchester in his *Treatise on the Management of Pregnant and Lying-in Women* (1773) asked that special attention should be devoted to all such matters as good hygienic surroundings, scrupulous cleanliness both personal on the part of all attendants and also of any material likely to come into contact with the patient, as bed linen, towels, sponges, instruments: proper ventilation of the labour room, maintenance of an even moderate room temperature and correct posture of the patient. As a result of his methods he reported that of the large number of lying-in patients he had delivered, he had never lost one from puerperal fever. Similar good results of White's methods were reported from Dublin. In 1841 Storrs of Sheffield, from his own observations and from evidence collected among his friends came to the conclusion that (1) puerperal fever may be communicated by touch and (2) that it originates in some animal poison, especially from erysipelas and its complications and in a lesser degree from typhoid fever. He therefore drew up the following rules for the obstetrician: (1) to avoid treating cases of erysipelas and (2) to avoid post-mortem examinations. If either or both of these procedures are unavoidable, then the practitioner should use disinfectants and change his clothes. Thus, disinfection measures against puerperal fever were already used in Great Britain and Ireland before Semmelweis' calls of warning.

Dr. Arneth, an assistant in the Viennese Obstetrical Clinic for midwives, a friend of Semmelweis and one of his earliest disciples, wrote in 1847 to the most famous obstetrician and gynaecologist of the time, James Young Simpson in Edinburgh and asked for his opinion. Simpson mistook the theory of Semmelweis for the English doctrine of contagion and was caustic in his reply. Later Simpson was made familiar with Semmelweis' views by Arneth in person and his reception was tolerant and dignified, while at the same time he put forward his own idea of the cause of puerperal fever. Simpson thought, that generally, if not always, the material which, when carried from one subject to another, could produce puerperal or surgical fever in a newly inoculable subject, was an *inflammatory secretion*, like the inoculable matter of small-pox, cow-pox, syphillis etc. Obstetricians had now a very decided proof of various kinds of morbid matters which were capable, when inoculated into the vagina of leading to puerperal fever. Simpson later abandoned the opinion that puerperal fever is a specific contagious disease and considered it to be identical with surgical fever. "The fever" he says, "is not the cause of the accompanying inflammation but the fever as well as the inflammations are the result of a common cause, namely the original corruptions of the blood. But what causes the corruption of the blood must be answered in a later era with a more developed pathological anatomy, histology and chemistry". Semmelweis answered Simpson by saying: "By Pyaemia I understand a disintegration of the blood, brought about by the introduction of a decomposed animal-organic matter and I call puerperal fever a variety of pyaemia".

We see the confusion that reigned in those days, when almost everybody anticipated the true essence of infection and yet: nobody knew.

Semmelweis was not a great writer. He published few papers, but in 1861 appeared his book: *The aetiology, conception and prophylaxis of puerperal fever*. In 1884 a text-book of gynaecology was published by Fritsch, then professor of gynaecology and obstetrics in Breslau; he referred to Semmelweis in these words: "In the history of midwifery there is a dark page, and it is headed: *Semmelweis*. What man could close his eyes to the powerful impression of his book? Even now there are whole pages of his deductions which might stand in the most modern work. And the annihilating logic of his statistics! We younger men, for whom antipathies are unthinkable, to whom the reading of coarse tirades about "genius misunderstood" are merely tedius, often find it incomprehensible that the logical conclusions to the doctrine infection were nowhere drawn: I refer to the local treatment. The efficient application of disinfection in midwifery is without doubt due to surgery yet most certainly it should have been the reverse. If the councils of Semmelweis had been followed, the truth of his doctrine would have been demonstrated in the compelling language of statistics, and so perhaps obstetrics would have stood in the forefront of the greatest advance in medicine of all time".

Semmelweis was not first to realize the devastations caused by puerperal fever, but none of his predecessors nor contemporaries had posed the problem of puerperal sepsis as clearly as he, for Semmelweis this was not only the fulfillment of his professional duties; it was a mission. Two discoveries at the beginning of this era of medicine had been of the highest importance in saving of life and preventing suffering: the discovery of Edward Jenner and that of Ignaz Semmelweis. In neither case did the discovery fall from heaven; in neither case was there a grasping of Promethean fire; for neither can one speak of inspiration. The discovery of Semmelweis was possible only for one who had prolonged and laborious preparation, who had directly observed and had reflected without preconceptions, whose intellect was alert and keen by reason of his human sympathy. The magnitude of his service to mankind on the one hand and his sufferings from jealousy, ignorance and ingratitude from his contemporaries on the other hand were extraordinary. The methods used against him to belittle and to destroy him were of a unique variety, intensity and extensiveness. When I have sometimes asked people whether they know or have heard of the name of Semmelweis the answer has been "Oh yes, but I cannot remember what he was or did"; or not infrequently "Never heard of him"!

But I am sure many of you will agree that he deserves to be remembered as one of the great personalities of the guild of Hippocrates.

